# Reducing the gender gap on adolescents’ interest in study fields: The impact of perceived changes in ingroup gender norms and gender prototypicality

**DOI:** 10.1007/s11218-024-09909-z

**Published:** 2024-04-04

**Authors:** Vincenzo Iacoviello, Giulia Valsecchi, Matthieu Vétois, Juan M. Falomir-Pichastor

**Affiliations:** FPSE, Université de Genève, Uni Mail, Boulevard du Pont d’Arve 40, 1205 Geneva, Switzerland

**Keywords:** Adolescents, Gender norms, Prototypicality, Education, Career choices

## Abstract

Despite some progress towards gender equality in Western societies, traditional gender norms still shape career choices, perpetuating a gender gap where girls are more likely to pursue traditionally feminine fields like healthcare, elementary education, and domestic roles (HEED), while boys are drawn to masculine domains such as science, technology, engineering, and mathematics (STEM). This research investigates whether, and under which conditions, the perception that gender norms are progressively changing towards less gender dichotomy can reduce this gender gap in academic fields. We recruited a sample of 642 high-school students (394 women and 248 men), and experimentally manipulated both the salience of changes in gender norm (stability vs change) and participants’ gender prototypicality. The main dependent variable was participants’ interest in stereotypically feminine (HEED) and masculine (STEM) academic fields. The results indicated a slight decrease in the gender gap for stereotypically feminine fields (HEED) among participants who saw themselves as typical members of their gender group, but no significant change was observed for stereotypically masculine fields (STEM). These findings suggest that shifting perceptions of gender norms may have a limited effect on modifying traditional educational and career choices, underscoring the resilience of entrenched gender stereotypes.

**Supplementary Information:**

The online version contains supplementary material available at 10.1007/s11218-024-09909-z.

## Introduction

From the end of WWII and under the impulse of feminist movements, Western societies have become more egalitarian. However, significant gender disparities (gender gap) persist in various domains. For instance, as compared to women, men continue to earn higher salaries (European Institute for Gender Equality, 2019), are more active in the labor force (United Nations, n. d.) and contribute less to housework (Gartland, 2017). As a matter of fact, progress towards gender parity in these domains seems to have stalled (Barroso & Brown, 2021). Existing literature suggests that traditional gender norms play a pivotal role in perpetuating gender inequality, subtly reinforcing the gender hierarchy (Burgess & Borgida, 1999; Connell & Messerschmidt, 2005).

Traditional gender norms encompass generalized beliefs about the typical roles and expectations associated with women and men based on their gender. These norms indicate that women are typically expected to exhibit relational traits such as warmth, compassion, and caregiving, while men are expected to display agentic traits like independence, autonomy, and determination (Eagly & Karau, 2002). These enduring expectations have a profound influence in people’s choices and behaviors, often starting at a young age (Aelenei et al., 2017; Buser et al., 2012). For instance, the gender-based categorization of toys leads children to prefer toys that align with their gender’s typical roles (Azmi et al., 2021; Davis & Hines, 2020). Moreover, traditional gender norms shape individuals’ future educational and career decisions, pushing young people toward fields that match the expected roles of their gender groups (Hegewish & Hartmann, 2014). This results in girls being drawn to HEED (health care, elementary education, and domestic) fields and boys gravitating toward STEM (science, technology, engineering, and mathematics) fields (Croft et al., 2015; Tellhed et al., 2018; see Su et al., 2009, for a meta-analysis). As society tends to accord greater prestige to stereotypically masculine domains over stereotypically feminine ones, traditional gender norms not only perpetuate gender disparities, but also reinforce the existing gender hierarchy (Hegewish & Hartmann, 2014).

Despite the robustness and durability of these processes, challenges to gender inequality and the dominance of masculinity over femininity are gaining social traction, resulting in shifts in gender norms and roles. However, to the best of our knowledge, few researches has investigated how these changes might influence young people’s career preferences (e.g., Cheng et al., 2020). This is somewhat surprising, given that gaining a better understanding of the potential consequences of these perceived changes in gender norms, as well as the underlying psychological processes at play, could significantly enhance efforts to reduce gender inequality. To fill in this gap in the literature, the main goal of the present research was to investigate whether and under what conditions perceived changes in gender norms influence gender disparities in chosen fields of study among young individuals. More specifically, we examined the moderating role of gender prototypicality.

### Perceived changes in gender norms

According to seminal theories on gender (e.g., social role theory, Eagly, 1987; gender schema theory, Bem, 1981), widely held beliefs and expectations regarding gender groups, encompassing personality traits, preferences, appearance and behavior, are rooted in the distribution of women and men into social roles. Consequently, women are both perceived and expected to be more communal and less agentic than men. This divergence arises because women are overrepresented in professions requiring communal attributes (e.g., social worker, teacher), while men are predominantly found in professions requiring agentic qualities (e.g., engineer, scientist).

Nevertheless, despite contextual and cultural variations, Western societies are witnessing modest yet noteworthy changes in the expected behaviors of boys and girls (Lopez Zafra & Garcia-Retamero, 2011). For instance, the influence of traditional gender norms has diminished over the course of the twentieth century, especially for traits typically associated with femininity (Bhatia & Bhatia, 2021). Relatedly, individuals are increasingly engaging in counter-stereotypical roles. For instance, more men are pursuing careers in nursing, and more women are entering fields like engineering, although this trend is less pronounced among men (Croft et al., 2015). Consequently, it is plausible to anticipate that the perception of an increase in counter-stereotypical behaviors, such as a higher prevalence of men in typically female professions (e.g., social workers) or of women in typically male professions (e.g., engineers), would challenge stereotypical social expectations and lead to reduced conformity with traditional gender norms.

However, recent research examining how the influence of perceived changes in gender norms on individuals’ behaviors have shown a more complex picture. Studies involving adults have revealed that perceived changes in gender norms do not consistently translate in reduced gender dichotomy. While some studies showed that perceived normative changes indeed motivate individuals to conform to new gender norms (i.e., a *conformity effect*, Borinca et al., 2021; Kosakowska-Berezecka et al., 2016; Valsecchi et al., 2023), other studies have revealed that perceived changes in gender norms actually increase defensive reactions aimed at protecting the traditional gender dichotomy (i..e, a *defensive reaction effect*; Falomir-Pichastor et al., 2019; Iacoviello et al., 2020).

Nonetheless, these seemingly contradictory effects are not necessarily incompatible. Indeed, subsequent research has shown that both effects come into play but are contingent on the individuals involved. First, Bosson and Michniewicz (2013) showed that this defensive reaction tends to be predominantly observed among men, while women tend to react positively to these perceived changes and show greater conformity to new, non-traditional gender norms. Second, research also indicates that men’s responses to perceived changes in gender norms vary depending on the degree to which they endorse traditional masculinity norms. On the one hand, less traditional men (i.e., those who endorse traditional masculinity norms to a lesser extent) are more likely to respond positively to perceived changes in gender norms and then conform more to the emerging non-traditional norms. These men tend to react to these perceived changes by diminishing stereotypically masculine self-descriptions (Babl, 1979), displaying reduced concern about being misclassified as gay (Borinca et al., 2021), and forming more favorable evaluations of non-traditional men (Iacoviello et al., 2021). On the other hand, more traditional men (i.e., those who strongly endorse traditional masculinity norms) are more prone to show a defensive reaction effect, thereby reaffirming traditional gender norms. In response to perceived changes in ingroup gender norms, these men tend to enhance stereotypically masculine self-descriptions (Babl, 1979), display heightened sexual prejudice (Falomir-Pichastor et al., 2019; Iacoviello et al., 2020; Valsecchi et al., 2022), and exhibit backlash against non-traditional men (Iacoviello et al., 2021).

In sum, the perception of changes in gender norms decreases conformity to traditional gender norms among women in general and less traditional men, while it strengthens conformity to these traditional gender norms among more traditional men. Despite the relevance of these findings for young people’s career aspirations, to our knowledge, there is currently no research investigating how adolescents react to perceived changes in their gender norms and, specifically, how these perceived changes influence their interest in gender-matching and non-matching study fields.

### Gender norms and adolescence

Adolescents’ self-concept is an evolving progress marked by changes in identity that occur throughout adolescence (Klimstra et al., 2010). It is characterized by fluctuations and a lack of consistency, making it challenging for adolescents to precisely define themselves (Harter, 1990; Rosenberg, 1986). As a result, they are particularly concerned with how others perceive them and are more susceptible to social influence compared to adults (Chein et al., 2011; Chierchia et al., 2020; Crone & Fuligni, 2020; Knoll et al., 2015). Accordingly, adolescents’ responses to perceived changes in gender norms may reflect a conformity effect, indicating a greater influence of emerging, non-traditional gender norms, which would suggest a stronger influence of traditional gender norms.

This assumption is supported by the literature on the influence of counter-stereotypical role models, which represent a small part of broader changes in gender norms. When adolescents observe individuals who defy traditional stereotypes, they may come to see these behaviors as normal and consider emulating them. Research in this area has predominantly focused on girls, demonstrating that female role models who have succeeded in non-stereotypical domains can inspire young girls to pursue similar paths (for a review, see Olsson & Martiny, 2018). For instance, the visibility of female politicians has been linked to increased intentions among adolescent girls to become politically active (Campbell & Wolbrecht, 2006). Likewise, exposure to female role models increases young girls’ aspirations for careers in STEM fields (González-Pérez et al., 2020). Similarly, when young girls learn about the growing representation of women in STEM fields, they express greater motivation to pursue a career in these areas (Cheng et al., 2020).

Furthermore, research on pluralistic ignorance shows that individuals might privately reject a certain belief while mistakenly assuming that the majority accepts it. This misunderstanding often leads them to conform publicly to what they perceive as the prevailing norm. In the context of traditional masculinity norms, these may persist artificially because men and boys might not be aware that many of their peers are also influenced by emerging, more progressive norms. An illustrative study by Kilmartin et al. (2008) demonstrates this phenomenon, showing that young men believed their peers endorsed more sexist beliefs than they privately reported. However, upon learning that their peers did not support these outdated norms to the extent assumed, these individuals distanced themselves from traditional forms of masculinity and embraced more progressive ones, resulting in a reduction in sexist attitudes.

Together, these findings suggest that adolescents, irrespective of their gender, are likely to conform to emerging gender norms rather than react defensively and reaffirm traditional ones. However, in the present research we also contend that conforming to newly established gender norms is not an automatic process; it actually requires specific psychological resources related to one’s self-concept.

### Gender prototypicality as a resource to conform to emerging norms

Conforming to norms play a pivotal role in shaping one’s identity (e.g. Smith et al., 2015; Tajfel & Turner, 1979). In contexts marked by normative changes, there’s often a co-existence of older, deeply ingrained norms and emerging, potentially conflicting ones (Iacoviello et al., 2022). Navigating such normative complexity or conflict can be challenging (see Allport, 1954). Given the deep-rooted nature of traditional gender norms in society, embracing an emerging norm may appear risky and unpredictable. For instance, men and women often face backlash when they display counter-stereotypical behaviors (Rudman et al., 2012). Therefore, conforming to new norms becomes more feasible when individuals perceive they still conform, to some extent, to the older norm. In other words, we posit that perceived group *prototypicality* (i.e., the extent to which individuals view themselves as representative exemplars of their ingroup) enables greater flexibility in conformity to changing norms.

Individuals often conform to ingroup norms to position themselves as exemplary prototypes of their group (Reicher et al., 2021; Turner, 1991). This conformity, in turn, bolsters acceptance within the group (Assilaméhou & Testé, 2013; Marques et al., 1988). Therefore, a secure sense of self emerges when people perceive themselves as prototypical members of their ingroup because they feel more integrated in it (Iacoviello & Spears, 2022; Leary, 2005). This sense of belonging, in turn, provides individuals with resources (or credentials) that afford them greater flexibility in how they align with the group norm. For instance, prototypical members are more likely to deviate from the group norms when they believe these norms are harmful for the group (Masson & Fritsche, 2019). These findings are consistent with self-licensing in conformity, which suggests that members who initially adhere to the egalitarian ingroup norm acquire ‘moral credentials’, allowing them to later deviate from this norm (e.g., Falomir-Pichastor et al., 2018; see also Mullen & Monin, 2016, for a review on self-licensing). Similarly, the gender literature indicates that men who are led to perceive themselves as typical rather than atypical members of their gender ingroup tend to show weaker adherence to traditional masculinity norms (Bosson et al., 2012).

Accordingly, we contend that focusing adolescents on their gender-typical traits and behaviors can foster a secure self-concept, shielding them from potential negative consequences when deviating from traditional gender norms. Thus, perceived gender prototypicality should ease adolescents’ conformity to alternative, emerging gender norms. Conversely, emphasizing their gender-atypical traits and behaviors would threaten their ingroup prototypicality, dissuading them from adopting emerging gender norms. To investigate this assumption, we experimentally manipulated participants’ ingroup prototypicality with respect to their gender group—namely the degree to which they are led to perceive themselves as gender-typical or gender-atypical exemplars of their gender group. We expected that adolescents who focus on situations where they exhibited gender-typical behavior should experience increased self-assurance and, paradoxically, a greater inclination to align with emergent and not-traditional gender norms.

## The present research

The present research aimed to examine the gender gap in chosen study fields among adolescents from Switzerland. A more central goal was also to investigate whether perceived changes in gender norms and one’s level of perceived gender prototypicality shape this gender gap. To do that, we recruited participants from two high schools in Geneva (i.e., Collège André-Chavannes and Collège Calvin), and asked them to fill in an online questionnaire. The study involved two key experimental manipulations. First, we experimentally manipulated the salience of gender ingroup norms by priming participants with either the stability of traditional gender norms (normative stability condition) or recent changes in gender norms (normative changes condition). Second, we experimentally manipulated gender prototypicality by prompting participants to reflect on either their gender-typical behaviors (gender-typical condition) or their gender-atypical behaviors (gender-atypical condition). We also introduced a control condition, where participants did not engage in any specific task.

Our main dependent variable was participants’ interest in fields stereotypically associated with femininity and masculinity. According to the revised literature we predicted that the gender gap in academic fields would decrease when changes (vs stability) in gender ingroup norms are made salient, specifically when participants are led to think about themselves as typical members of their gender ingroup. In sum, we hypothesized that the reduction of the gender gap in normative changes condition should be observed in the gender-typical condition, but not (or less so) in both the gender-atypical and control conditions. For a more comprehensive analysis, we separately formulated this hypothesis for each type of field: stereotypically feminine fields (H1) and masculine fields (H2).

### Method

#### Participants and procedure

We recruited participants from two high schools in Geneva, Switzerland. In Geneva, students typically enter high school at the age of 15 and graduate at 19. To recruit participants who still had flexibility in choosing their future careers and would therefore be more responsive to the experimental inductions, we only recruited students at the beginning of their high school curriculum. This included all first- and second-year students form the two institutions. Initially, 688 students agreed to participate. After removing 46 participants namely because lack of consent (*n* = 38) and diligence (*n* = 8)^1^, the final sample consisted of 642 (61.4% female, 38.6% male, *M*_age_ = 15.57 years, *SD* = 0.83). A majority of participants (84.9%) reported being attracted to people of the opposite sex only, 9.5% to both sexes, 3.4% to people of the same-sex only, and 0.2 to neither (2.0% did not answer). We conducted a sensitivity power analysis using G*Power for a multiple linear regression model with 11 predictors, including main effects and interactions (α = 0.05, two-tailed; power = 0.80). This analysis revealed that our final sample (*N* = 642) was adequately powered to detect a small effect size (η^2^ = 0.01).

#### Procedure

Participants took part in the study by completing an online questionnaire. They were accompanied by their head teacher into a computer room, where an experimenter introduced them to the study. While most participants completed the study on computers, a minority (8%) used their smartphone. Initially, participants provided information about their gender and sexual orientation, as mentioned earlier. Next, the questionnaire presented the manipulation of the salience of the gender ingroup norm, followed by the manipulation of the gender prototypicality. Subsequently, participants indicated their interest in stereotypically feminine and masculine study fields before providing demographic information^2^.

All participants, whether they completed the whole questionnaire or quitted prematurely, were directed to a debriefing page that explained the purpose of the study. Finally, at the end of each session, the experimenter conducted a debriefing with participants. After the debriefing, participants were asked to provide their consent for the use of their data. This study was approved by the ethical committee of the authors’ institution (approval N° PSE.20200303.06), and data are available on OSF (https://osf.io/3v5f2/?view_only=8a424c69f3fe4fec8401db1bfc633f93).

#### Independent variables

##### Manipulation of the gender-related ingroup norm

Existing research often employs deception to manipulate changes in the gender norms (e.g., Falomir-Pichastor et al., 2019), the present study aimed to avoid deceiving adolescent participants. Instead, we manipulated gender norms by directing participants’ attention to specific aspects of these norms. In the “salience of change” condition, participants were prompted to consider the evolution of the ingroup norm over time, while in the “salience of status quo” condition, participants were directed to focus on the existing discrepancies between men and women. Participants read a short text about the purported results of studies, and the content of this text varied based on the participants’ gender. For female participants in the *salience of change* condition, the text stated: “These studies showed that most women tend to adopt a more masculine lifestyle in their life choices and professional activities. Indeed, there is a growing number of women participating in activities (e.g., football) and pursuing careers (e.g.,, firefighter) traditionnally associated with men”. In contrast, in the *salience of status quo* condition, female participants were informed that “These studies showed that most women are still embrace a very feminine lifestyle in their choices and professional activities. Indeed, a majority of women continue to engage in activities (e.g., dancing) and professions (e.g., nurse) traditionnally associated with women”. For male participants, the content of the articles was identical but reversed, discussing changes in lifestyle, study and professional activities among boys. To reinforce the manipulation, participants were further informed that “These findings certainly resonated with their personal experiences”, and they were invited to provide a short example illustrating these study findings in an open-ended question.

##### Manipulation of gender prototypicality

To manipulate gender prototypicality we adapted the induction used by Bosson and Michniewicz’s (2013; Study 4) that focused participants on their level of conformity to gender norms. In the *gender-typical* condition, boys [girls’ version in brackets] were asked to: “think about a time in your life when you felt you were behaving as a ‘real man [woman]’ and others recognized you as such”. In the *gender-atypical* condition, boys [girls’ version in brackets] were asked to: “think about a time in your life when you did not feel you were behaving as a ‘real man [woman]’ and others did not recognize you as such”. Participants were also informed that: “if you cannot recall such a time right now, you can imagine a hypothetical situation in which this might have occurred”. In both conditions, participants were instructed to describe the specific situation they had in mind. In the *control condition*, participants proceeded directly to respond to the dependent variables immediately after the salience of normative changes manipulation. By including this control condition as an additional comparison, we aimed to bolster our confidence that any observed effects were specifically attributable to gender prototypicality itself, rather than being influenced by a third variable related to experimental demands or the act of engaging in introspective tasks.

#### Measure of interest in study fields

To assess participants’ interest in various study fields, we asked them rate their interest in seven stereotypically feminine fields (i.e., psychology, art history, geography, general history, translation and interpretation, educational sciences, languages and literature) and seven stereotypically masculine fields (i.e., physic, astronomy, informatic, mathematic, chemistry and biochemistry, engineering sciences, architecture). These fields were selected based on their gender ratio at the local university [i.e., University of Geneva] and other higher-education institutions. Participants rated their interest in a 7-point scale, ranging from “not interested at all” (1) to “very interested” (7).

To ensure that participants perceived these fields as distinct categories, we performed a principal component analysis with varimax rotation. The results indicated the existence of two factors, with a clear separation between stereotypically masculine and feminine fields. Six out of seven stereotypically masculine fields (physic, astronomy, informatic, mathematic, chemistry and biochemistry and engineering sciences) loaded positively (loadings > 0.55) on the first factor (λ = 3.18), and all seven stereotypically feminine fields loaded positively (loadings > 0.44) on the second factor (λ = 2.93). Architecture showed moderate loadings on both dimensions (0.27 and 0.26). Moreover, the inclusion of architecture slightly weakened the reliability of the stereotypically masculine fields (α = 0.78, compared to α = 0.80 when excluded). Thus, architecture was excluded from the final score of stereotypically masculine fields (*M* = 3.95, *SD* = 1.46). The reliability of the seven stereotypically feminine fields was satisfactory (α = 0.74, *M* = 3.75, *SD* = 1.20) and was not improved by excluding any of the fields.

### Results

Given the complexity of the experimental design, we used contrast analyses (instead of omnibus tests) to test the two hypotheses. Contrast analysis is particularly suitable when dealing with variables having more than two modalities, as it allows for testing specific hypotheses without arbitrarily dividing variance effects (see Brauer & McClelland, 2005; Furr & Rosenthal, 2003). Therefore, for the gender prototypicality variable, we computed two orthogonal Helmert contrasts from the three conditions. The first contrast (C1) compared the gender-typical condition (coded + 2) against both the control and gender-atypical conditions (both coded − 1). To examine the residual effect, we created a second contrast (C2) where the control condition was coded − 1, the gender-typical condition was coded 0 and the gender-atypical condition was coded + 1. The residual variance should account for only a non-significant part of the total variance. According to the main hypotheses, we expected the overall interaction involving C1 to be significant, while the interaction involving C2 should not. Subsequently, we conducted two separate linear regressions—one for the interest in stereotypically feminine study fields and another for interest in stereotypically masculine study fields. The predictors included participant gender (coded − 1 for female and + 1 for male), salience of gender-related ingroup norm (coded − 1 for stability and + 1 for change), C1, C2, and their interactions (except those that included the two orthogonal contrasts). Results of the two linear regressions analyses are displayed in Tables 1 and 2.

**Table 1:** Results of the linear regression on interest in stereotypically feminine study fields

	*b*	*t*	*p*	95% CI		η_p_^2^
				LB	UB	
Intercept	3.69	77.65	< .001	3.59	3.78	0.91
Participant gender (PG)	− 0.27	− 5.65	< .001	− 0.36	− 0.18	0.05
Ingroup Norm (IN)	0.06	1.34	.180	− 0.03	0.16	< 0.01
C1	0.04	1.15	.249	− 0.03	0.11	< 0.01
C2	− 0.10	− 1.73	.085	− 0.21	0.01	0.01
PG × IN	0.03	0.67	.506	− 0.06	0.13	< 0.01
PG × C1	0.04	1.13	.257	− 0.03	0.11	< 0.01
IN × C1	− 0.05	− 1.40	.163	− 0.11	0.02	< 0.01
PG × C2	− 0.13	− 2.29	.023	− 0.25	− 0.02	0.01
IN × C2	− 0.00	− 0.06	.955	− 0.12	0.11	< 0.01
PG × IN × C1	0.07	2.02	.044	0.00	0.14	0.01
PG × IN × C2	0.02	0.31	.757	− 0.10	0.13	0.00

**Table 2:** Results of the linear regression on interest in stereotypically masculine study fields

	*b*	*t*	*p*	95% CI		η_p_^2^
				LB	UB	
Intercept	4.04	70.53	< .001	3.93	4.15	0.89
Participant gender (PG)	0.40	6.90	< .001	0.28	0.51	0.07
Ingroup Norm (IN)	0.04	0.72	.471	− 0.07	0.15	< 0.01
C1	0.00	0.08	.940	− 0.08	0.08	< 0.01
C2	− 0.08	− 1.09	.276	− 0.21	0.06	< 0.01
PG × IN	0.09	1.52	.130	− 0.03	0.20	< 0.01
PG × C1	0.06	1.50	.135	− 0.02	0.14	< 0.01
IN × C1	0.00	0.04	.970	− 0.08	0.08	< 0.01
PG × C2	− 0.01	− 0.08	.934	− 0.14	0.13	< 0.01
IN × C2	0.10	1.50	.135	− 0.03	0.24	< 0.01
PG × IN × C1	− 0.03	− 0.77	.442	− 0.11	0.05	< 0.01
PG × IN × C2	0.08	1.09	.277	− 0.06	0.21	< 0.00

#### Interest in stereotypically feminine study fields

**Fig. 1:**
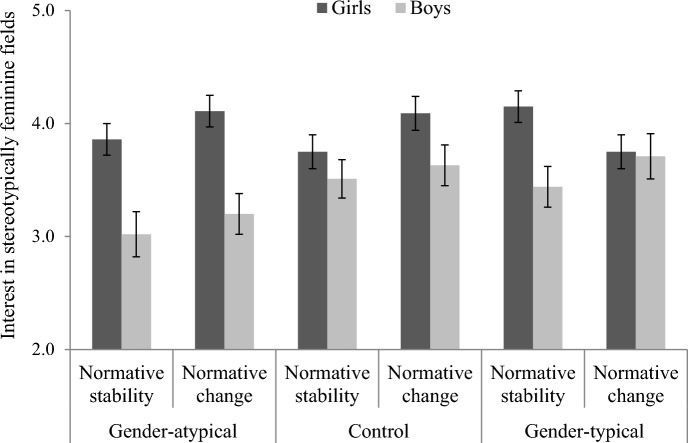
Girls’ and boys’ interest in stereotypically feminine fields as a function of salience of changes in gender norms and gender prototypicality. Error bars represent standard errors

The analysis revealed a main effect of participant gender, *B* = − 0.27, *SE* = 0.05, *t*(630) = − 5.65, *p* < .001, 95% CI [ − 0.36, − 0.18], η_p_^2^ = 0.05. Overall, female participants reported greater interest in stereotypically feminine fields than male participants (respectively, *M* = 3.95, *SE* = 0.06, and *M* = 3.42, *SE* = 0.07). This effect was further qualified by a significant participant gender × gender-related ingroup norm × C1 interaction, *B* = 0.07, *SE* = 0.03, *t*(630) = 2.02, *p* = .044, 95% CI [0.00, 0.14], η_p_^2^ = 0.01. Specifically, in the modality combining the control and gender-atypical conditions, the simple effect of gender was not dependent on the salience of the gender-related ingroup norm, *B* = − 0.04, *SE* = 0.06, *t*(630) = − 0.76, *p* = .446, 95% CI [− 0.16, 0.07], η_p_^2^ < 0.05. Furthermore, and in line with H1, the participant gender × gender-related ingroup norm interaction was significant in the gender-typical condition, *B* = 0.17, *SE* = 0.08, *t*(630) = 2.01, *p* = .045, 95% CI [0.00, 0.33], η_p_^2^ = 0.01. The decomposition of this interaction revealed that the effect of gender was significant in the stability condition (*M*_girls_ = 4.15, *SE*_girls_ = 0.14; *M*_boys_ = 3.44, *SE*_boys_ = 0.18), *B* = − 0.36, *SE* = 0.11, *t*(630) = − 3.18, *p* = .002, 95% CI [ − 0.58, − 0.14], η_p_^2^ = 0.02, but not in the change condition, (*M*_girls_ = 3.75, *SE*_girls_ = 0.15; *M*_boys_ = 3.71, *SE*_boys_ = 0.20), *B* = − 0.02, *SE* = 0.12, *t*(630) = − 0.19, *p* = .851, 95% CI [ − 0.27, 0.22], η_p_^2^ < 0.01. This significant reduction in the gender gap results from a combined trend observed both for female and male participants. However, the effect of gender-related ingroup norm was neither significant for male participants, *B* = 0.14, *SE* = 0.13, *t*(630) = 1.04, *p* = .300, 95% CI [ − 0.12, 0.40], η_p_^2^ < 0.01, nor female participants, *B* = − 0.20, *SE* = 0.20, *t*(630) = − 1.96, *p* = .050, 95% CI [ − 0.40,  − 0.00], η_p_^2^ = 0.01 (see Fig. 1).

Finally, the analysis also revealed a significant gender × C2 interaction, *B* = − 0.13, *SE* = 0.06, *t*(630) = − 2.29, *p* = .023, 95% CI [ − 0.25,  − 0.02], η_p_^2^ = 0.01. Specifically, the gender gap was greater in the gender-atypical condition (*M*_girls_ = 3.92, *SE*_girls_ = 0.11; *M*_boys_ = 3.57, *SE*_boys_ = 0.12), *B* = − 0.88, *SE* = 0.17, *t*(630) = − 5.32, *p* < .001, 95% CI [ − 1.20, − 0.55], η_p_^2^ = 0.04, than in the control condition (*M*_girls_ = 3.99, *SE*_girls_ = 0.10; *M*_boys_ = 3.11, *SE*_boys_ = 0.13), *B* = − 0.35, *SE* = 0.16, *t*(630) = − 2.14, *p* = .033, 95% CI [ − 0.67, − 0.03], η_p_^2^ = 0.01. All other effects were not significant, all *ps* > .085, including the participant gender × gender norm × C2 interaction, *B* = 0.02, *SE* = 0.06, *t*(630) = 0.31, *p* = .757, 95% CI [ − 0.10, 0.13], η_p_^2^ < 0.01.

#### Interest in stereotypically masculine study fields

**Fig. 2:**
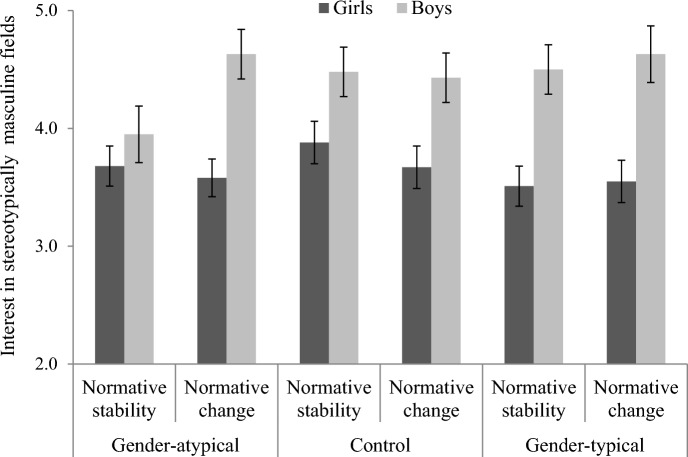
Girls’ and boys’ interest in stereotypically masculine fields as a function of salience of changes in gender norms and gender prototypicality. Error bars represent standard errors

We conducted a similar linear regression analysis on the interest in stereotypically masculine fields. The results only revealed a significant main effect of gender, *B* = 0.40, *SE* = 0.06, *t*(630) = 6.90, *p* < 0.001, 95% CI [0.28, 0.51], η_p_^2^ = 0.07. Specifically, male participants reported significantly higher interest in stereotypically masculine fields (*M* = 4.44, *SE* = 0.09) compared to female participants (*M* = 3.65, *SE* = 0.07). At odds with H2, the participant gender × gender-related ingroup norm × C1 interaction was not significant, *B* = − 0.03, *SE* = 0.04, *t*(630) = − 0.77, *p* = 0.442, 95% CI [ − 0.11, 0.05], η_p_^2^ < 0.01. Similarly, the participant gender × gender-related ingroup norm × C2 interaction also did not reach significance, *B* = 0.08, *SE* = 0.07, *t*(630) = 1.09, *p* = 0.277, 95% CI [ − 0.06, 0.21], η_p_^2^ < 0.01 (see Fig. 2). All other effects were not significant, all *ps* > 0.130.

## Discussion

This study tested the hypothesis that perceiving gender norms as evolving towards less pronounced gender differences would lead adolescents of both genders to exhibit more similar interests in study fields (i.e., narrowing the gender gap), provided they can perceive themselves as prototypical exemplars of their gender group. Furthermore, we expected this effect in both stereotypically feminine (H1) and masculine (H2) fields. Overall, the results only provided partial support for the general hypothesis, indicating a complex interplay between gender norm perceptions, gender prototypicality, and interests in academic fields.

More specifically, we observed a strong gender gap in both stereotypically feminine and masculine fields. Girls showed a stronger preference for feminine fields than boys, and boys showed more interest in masculine fields than girls. Importantly, the predicted reduction in the gender gap was observed in stereotypically feminine fields, but not in masculine ones. Specifically, perceiving gender norms as changing reduced the gender gap in stereotypically feminine fields only among adolescents who viewed themselves as typical representatives of their gender. This finding provides empirical support for Hypothesis 1, though the effect size was relatively small (η_p_^2^ = 0.01). However, the perception of changing gender norms did not influence the gender gap in stereotypically masculine fields, regardless of the manipulation of the gender prototypicality, providing no support for Hypothesis 2.

### Is the gender dichotomy inescapable?

The present findings highlight the robustness and persistence of the gender gap in study fields. Even among adolescents at the beginning of their high-school education, with a myriad of potential academic paths ahead of them, the gender gap in study preferences remained conspicuous, yielding medium-sized effects in both stereotypically feminine and masculine fields (η_p_^2^ = 0.05 and η_p_^2^ = 0.07, respectively). This suggests that despite the greater openness of the so-called generation Z towards issues of gender and sexual identity (Moscowitz et al., 2022; Phillips et al., 2019), this progressive mindset does not necessarily translate into personal interest in fields traditionally associated with the opposite gender. Accordingly, challenging deeply ingrained gender norms presents a complex task.

Notably, the trend toward gender equality in Western societies seems to have slowed down (Barroso & Brown, 2021). In some circumstances, gender-related issues like domestic violence against women have even intensified during crises such as the COVID-19 pandemic (Piquero et al., 2021). Moreover, countries that have made more substantial strides toward parity paradoxically exhibit greater gender segregation across different occupations (Stoet & Geary, 2018). Recent research has even suggested that this *gender-equality paradox* might be linked to more egalitarian and developed countries endorsing traditional gender beliefs, such as the idea that certain subjects like math are not suitable for girls, to a greater degree (Breda et al., 2020). Therefore, traditional gender norms continue to play a pivotal role in perpetuating gender disparities and impeding progress toward parity.

In this regard, the present findings offer a nuanced view that is both disappointing and hopeful. On the one hand, they might appear disappointing because, despite observing a significant reduction in the gender gap related to the salience of normative changes, the size of this effect was relatively small. Indeed, this reduced gap resulted from a combined slight increase in boys’ interest in feminine fields and a slight decrease in girls’ interest in these field, yet these changes were so minor that neither was statistically significant. The modest size of the reduced gap effect calls for caution regarding their robustness in the absence of replication attempts.

The significance of these findings is also limited by the fact that perceived changes in gender norms only influenced interest in stereotypically feminine fields when participants perceived they were typical representatives of their gender group. Without this sense of gender typicality, in both the control and gender-atypical conditions, the perceived changes in norms were not strong enough to reduce the gender gap. This aligns with research suggesting that deviating from traditional gender norms can lead to social penalties, such as social backlash and ostracism (Iacoviello et al., 2021; Rudman et al., 2012), even though the perceived risk of such consequences is likely overestimated (Kilmartin et al., 2008; Meimoun et al., 2023).

On the other hand, these results can nevertheless be seen as encouraging. Indeed, they showed that making normative changes salient can reduce the gender gap in the interest for stereotypically feminine fields, to the extent of making it statistically non-significant. This effect is noteworthy, especially considering the experimental procedure used, which was conducted in an environment far from ideal. Participants completed the online questionnaire in a classroom setting alongside their classmates. Therefore, the presence of peers was salient, and there were distractions as some students engaged in conversations and laughter despite interventions from the experimenter and teacher at times. This suggests that future research using more refined and controlled interventions based on normative changes (such as workshops, round table discussion, etc.) might yield stronger and more reliable effects.

A final theoretical consideration refers to the rationale behind our research and predictions. We reasoned that adolescents who are led to perceive themselves as prototypical members of their gender ingroup would possess a more secure self, equipping them with greater resources to be influenced by an emerging, more progressive norm. However, we have to acknowledge that an alternative, though compatible, explanation can be advanced. Indeed, according to self-categorization theory (Turner et al., 1987), fostering the perception of individuals as typical members of their gender group might heighten the salience of their ingroup or strengthen their identification with it. As a result, they would be more inclined to conform to the ingroup norm, even if it is an emerging one. Existing literature indeed indicates that conformity usually increases when the ingroup is salient (Abrams et al., 1990; Robertson, 2006) and when ingroup identification is high (Ellemers et al., 2002; Falomir-Pichastor et al., 2013; see Packer, 2008, for a review). Moreover, in contrast to atypical members, typical group members exhibit conformity behaviors that are not superficial or driven by self-interests, as their behaviors remain consistent regardless of the public or private context (Noël et al., 1995). In sum, the reduction of the gender gap observed in the normative changes condition may be attributed to fundamental dynamics related to self-categorization that are accentuated when adolescents are led to perceive themselves as typical ingroup members. Future research should delve deeper into investigating the specific conditions under which each of these two mechanisms is at work^3^.

### Limitations and future research

Although the present findings provide valuable insights, it is important to acknowledge certain methodological limitations. First, one important factor that mitigates the scope of the present research is the non-confirmation of the main hypothesis regarding stereotypically masculine fields (H2). Nevertheless, this result is in line with research showing that, between 1993 and 2012, women displayed a decreasing tendency to endorse stereotypically feminine traits over time, but did not exhibit a corresponding increase in the endorsement of stereotypically masculine traits (Donnelly & Twenge, 2017). Future research should investigate more in depth whether perceived normative changes are indeed less likely to manifest in traditionally masculine (as opposed to feminine ones) for both girls and boys.

Furthermore, in the gender-atypical condition, we asked participants to recall an event where they acted as “a real man/woman”. This procedure might have highlighted traditional gender norms (Bosson & Michniewicz, 2013). It is possible that asking adolescents who do not conform to traditional gender norms to recall a situation in which they acted as a ‘real man’ or ‘real woman’ could have been unsettling for them, which may have affected their subsequent answers.

It is also worth noting that in the text manipulating the salience of normative change versus stability, an example of either a traditionally feminine occupation (nurse) or a traditionally masculine occupation (firefighter) was cited. Both of these occupations might be seen as somewhat irrelevant, as they typically do not require a trajectory involving higher education. Rather than a weakness, we believe this actually strengthens the results we obtained for feminine fields. Despite the potential lack of direct relevance to these occupations, participants could still infer a general trend toward normative changes in gender norms, leading to a reduced gender gap. That being said, it is important to acknowledge that feminine and masculine occupations may not be equivalent in their relevance. Some of the students might indeed pursue a career as a nurse at the end of their education, while pursuing a career as firefighter is less likely. This difference could potentially explain why the results for masculine fields were not consistent with our hypothesis. In future research, it would be advisable to incorporate manipulations of normative changes that encompass a broader range of occupations, including scenarios where no specific occupations are mentioned.

Moreover, the present results suggest that conforming to new gender norms is a complex process of identity change within a context where various gender norms coexist, have different values, and compete with each other. In this context of normative complexity, participants paradoxically conformed to emerging and progressive gender norms when they were led to think about themselves as endorsing traditional gender norms (i.e., when they felt they were typical of their gender ingroup). These findings align with previous research indicating that men adapt to normatively complex contexts by conforming to either traditional or emerging masculinity norms based on the ingroup versus intergroup context (Iacoviello et al., 2022). However, it is important to note that while these findings provide some insights, they do not directly illustrate conformity processes in normatively complex contexts. Therefore, we strongly encourage future research to examine the processes of identity change related to adolescents’ conformity to gender norms in contexts characterized by social change and normative complexity.

Finally, our manipulation of the salience of normative changes was gender-specific: Boys read a text stating that men are changing, and girls read a text stating that women are changing. We chose to specifically manipulate changes in the ingroup gender norm because existing literature suggests that ingroup norms typically obtain more influence than outgroup norms (Jetten et al., 1996). However, it’s possible that a more general manipulation of societal changes, which encompasses both men and women, could yield stronger effects. Therefore, in future research, it would be worthwhile to investigate the effect of such broad manipulations of societal changes.

## Conclusion

Past research on the effect of perceived changes in gender norms has focused on adult samples, and the results indicate that these changes can sometimes trigger defensive reactions aimed at preserving traditional gender roles, particularly in men who strongly adhere to these norms and thus perceive changes as threatening. Our results on an adolescent sample suggest that young individuals, with their more malleable identities and openness to new norms, may be less resistant to these changes. However, the small effect size calls into question the robustness and generalizability of the present findings. Thus, this study underscores the importance of investigating the social consequences of evolving gender identities across different social groups. In order to achieve gender equality, it’s imperative to challenge entrenched gender norms. Educating the youth about the evolving nature of gender norms can help them recognize that what may have seemed counter-normative is increasingly becoming accepted as the new “norm”.

### Supplementary Information

Below is the link to the electronic supplementary material.

Supplementary file1 (PDF 116 KB)

## Footnotes

1 Two participants reported the unlikely age of 25 years old (which was 9.65 *SD* from the mean), 2 participants who participated in the same session provided the same answer to the open-ended question, and 4 participants provided irrelevant answers in the open-ended question which suggested they did not take the questionnaire seriously (instead of answering the question, they wrote a sentence reproducing a popular sexist French-speaking meme).

2 We hereby present all the relevant material to test our hypothesis. However, we included additional measures (i.e., traditional gender roles, ability beliefs and interest for foreign languages) for exploratory purposes and in order to meet educational goals (i.e., provide feedback to the institution and to the participants). Information related to these additional variables can be found in the supplementary material file.

3 We are grateful to an anonymous reviewer for suggesting this alternative explanation of the present findings.
