# Supplementary material for: Reducing the gender gap on adolescents’ interest in study fields: The impact of perceived changes in ingroup gender norms and gender prototypicality
Source: Soc Psychol Educ. 2024 Apr 4;27(3):1043–63. doi: 10.1007/s11218-024-09909-z (PMC11271416; doi:10.1007/s11218-024-09909-z)
Supplement: Supplementary file 1 — Supplementary file1 (PDF 116 KB) [file 11218_2024_9909_MOESM1_ESM.pdf]

Supplementary material

Traditional gender roles.

Participants' endorsement of traditional gender roles was measured at the beginning of the questionnaire (before the manipulations) to explore the participants' level of endorsement. Our 10-item scale was inspired from the Gender Role subscale of Morgan's (1996) Liberal Feminist Attitude and Ideology Scale (LFAIS). Sample items are: "A man's main obligation is to bring a salary back home" and "Both the man and the woman should take care of the children" (reverse coding). Participants responded on a 7-point scale ranging from "not agree at all" (1) to "completely agree" (7). We computed a score of endorsement of traditional gender roles such as a higher score indicates greater endorsement ($\alpha = .79$, $M = 1.86$, $SD = 0.80$).

In order to examine participants' endorsement of traditional gender roles, we looked at the descriptive statistics. First, the mean (1.86), the median (1.6) and the mode (1) were quite low (on a scale going from 1 to 7). Second, the distribution appeared to be positively skewed (skewness = 1.59) as it exceeded the range of values to be considered "normal" (i.e., between -1 and 1; Hair et al., 2017). These elements suggested a floor effect on the score of traditional gender roles, and therefore indicated that participants tended to reject traditional gender roles. This was however especially the case for girls ($M = 1.61$, $SE = 0.04$), as compared to boys ($M = 2.26$, $SD = 0.05$), $F(1,640) = 117.20$, $p < .001$, $\eta_p^2 = .16$.

Despite this limitation in the scale's properties, we ran a final analysis to examine if the effect predicted in H1 was still reliable will including traditional gender roles in the analysis. We therefore performed a linear regression on the score of interest for feminine fields, with participants' gender (coded −1 for female and +1 for male), salience of gender-related ingroup norm (coded −1 for stability and +1 for change), traditional gender roles

(centered continuous variable), C1, C2, and their interactions (except those including the two orthogonal contrasts). The participant gender × gender-related ingroup norm × C1 interaction remained significant, $B = 0.08$, $SE = 0.04$, $t(641) = 2.03$, $p = .043$, 95% CI [0.00, 0.15]. Moreover, the higher-order gender × gender-related ingroup norm × C1 interaction × traditional gender role interaction was not significant, $B = 0.04$, $SE = 0.05$, $t(641) = 0.81$, $p = .417$, 95% CI [-0.06, 0.13]. This showed that the pattern predicted by H2 was not dependent on participant's endorsement of traditional gender roles.

We performed the same analysis on the interest for masculine fields. Results showed that the higher-order gender × gender-related ingroup norm × C1 interaction × traditional gender role interaction was not significant, $B = -0.11$, $SE = 0.06$, $t(641) = -1.84$, $p = .066$, 95% CI [-0.22, 0.01], suggesting that the predicted in H2 was not dependent on participant's endorsement of traditional gender roles.

Ability beliefs

After they answered the interest items, participants were shown the same fourteen fields of studies and were asked to indicate their perceived ability to engage in these fields. Responses were collected on a 7-point scale going from "not capable at all" (1) to "very capable" (7). A principal component analysis showed that, on the first dimension ($\lambda = 3.50$), all the seven feminine fields loaded substantially and positively (loadings > .55). On the second dimension ($\lambda = 3.39$), six out of seven masculine fields (physic, astronomy, informatic, mathematic, chemistry and biochemistry and engineering sciences) loaded substantially and positively (loadings > .61). *Architecture* loaded moderately (.30 and .35) on both dimensions. It also decreased the reliability of the masculine fields (from $\alpha = .81$ to $\alpha = 83$ after exclusion). Therefore, *architecture* was excluded from the final score of masculine fields ($M = 4.15$, $SD = 1.42$). The reliability of the seven feminine fields was satisfying ($\alpha = .82$, $M = 4.45$, $SD = 1.28$), and was not increased by the exclusion of any of the fields.

In order to examine the impact of our independent variables on the ability beliefs, we performed two linear regressions on ability beliefs for both feminine and masculine fields. In both analyses, the predictors were participants' gender (coded −1 for female and +1 for male), salience of gender-related ingroup norm (coded −1 for stability and +1 for change), C1, C2, and their interactions (except those including the two orthogonal contrasts). The analysis on the feminine fields only showed a main effect of participant gender, $B = -0.33$, $SE = 0.05$, $t(641) = -6.59$, $p < .001$, 95% CI [-0.43, -0.23]. Female participants felt more able to engage in feminine fields than male participants (respectively, $M = 4.70$, $SE = 0.06$, and $M = 4.04$, $SE = 0.08$). All other effects were not significant, all *ps* > .100.

The analysis on the masculine fields also showed a main effect of participant gender, $B = 0.33$, $SE = 0.06$, $t(641) = 5.771$, $p < .001$, 95% CI [0.21, 0.44]. Male participants felt more able to engage in masculine fields than female participants (respectively, $M = 4.55$, $SE = 0.09$, and $M = 3.90$, $SE = 0.07$). Moreover, we observed an interaction between participant gender and C1, $B = 0.09$, $SE = 0.04$, $t(641) = 2.29$, $p < .023$, 95% CI [0.01, 0.17]. Specifically, the gender gap was greater in the prototypicality affirmation condition ($M_{boys} = 4.75$, $SE_{boys} = 0.16$, and $M_{girls} = 3.73$, $SE_{girls} = 0.12$) than in the modality combining the prototypicality threat and control conditions ($M_{boys} = 4.47$, $SE_{boys} = 0.11$, and $M_{girls} = 3.98$, $SE_{girls} = 0.09$). All other effects were not significant, all *ps* > .067.

Interest in foreign languages

After the ability beliefs' items, participants answered a 9-item scale adapted from Gardner et al. (1997). Sample items are: "I wish I could read newspapers and magazines in another language" and "I am not interested in learning a foreign language" (reverse coded). Participants responded on a 7-point scale going from "not at all" (1) to "completely" (7). After recoding, a score of interest in foreign languages was computed such as a higher score indicates a higher interest ($\alpha = .77$, M = 5.61, SD = 0.99).

The same linear regression analysis was performed on the interest in foreign languages. The results showed a main effect of participant gender, $B = -0.31$, $SE = 0.04$, $t(641) = -8.03$, $p < .001$, 95% CI [-0.39, -0.23]. Female participants indicated a greater interest in foreign languages than male participants (respectively, $M = 5.85$, $SE = 0.05$, and $M = 5.23$, $SE = 0.06$; see Figure 1). The main effect of C2 was also significant, $B = -0.12$, $SE = 0.03$, $t(641) = -2.47$, $p = .014$, 95% CI [-0.21, -0.02]. The interest in foreign languages was greater in the identity threat condition ($M = 5.43$, $SE = 0.07$) than in the control condition ($M = 5.66$, $SE = 0.07$). All other effects were not significant, all *ps* > .190.

Hair, J. F., Hult, G. T. M., Ringle, C. M., and Sarstedt, M. 2017. *A Primer on Partial Least Squares Structural Equation Modeling (PLS-SEM)*. 2nd Ed. Thousand Oaks, CA: Sage
